# Opportunities and challenges in China's CDC system reform: a grassroots perspective

**DOI:** 10.1093/inthealth/ihaf061

**Published:** 2025-05-30

**Authors:** Yanqing Zhang, Yongtao Zheng, Chunmei Ye, Biao Li

**Affiliations:** Xiaoshan District Center for Disease Control and Prevention, No. 3258, Fengqing Avenue, Xiaoshan District, Hangzhou 310000, China; Xiaoshan District Center for Disease Control and Prevention, No. 3258, Fengqing Avenue, Xiaoshan District, Hangzhou 310000, China; Linping District Center for Disease Control and Prevention, No. 930, Century Avenue, Linping District, Hangzhou 310000, China; Hangzhou Center for Disease Control and Prevention, No. 568, Mingshi Road, Shangcheng District, Hangzhou 310000, China

To the Editor,

The disease prevention and control system plays a crucial role in safeguarding public health, ensuring economic and social stability and mitigating emerging health threats. Since the establishment of China's first national health and epidemic prevention stations in 1953, the country's disease prevention and control system has been evolving for >7 decades (Figure 1). Between 1950 and 2010, disease prevention efforts contributed to >75% of the increase in life expectancy. However, the 21st century has brought new challenges, including an aging population, shifts in disease patterns and emerging infectious diseases. The coronavirus disease 2019 pandemic particularly underscored the need for structural reforms in China's public health system.^1^ In response, the National Bureau of Disease Control and Prevention was established in May 2021 to promote public health in a comprehensive manner, marking a major step forward in enhancing the nation's post-epidemic public health governance, and by December 2023, the Chinese government issued the Guiding Opinions on Promoting High-Quality Development of Disease Prevention and Control. Over the past year, local Centers for Disease Control and Prevention (CDC) and Health Supervision Institutes have been systematically merged, marking a new phase in China's public health governance.

**Figure 1.**
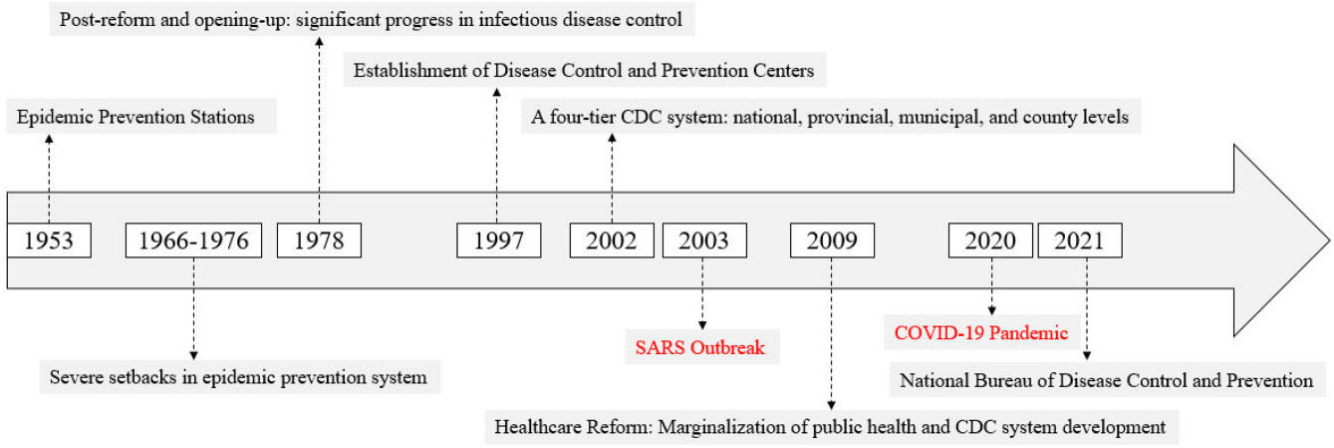
The development and reform process of China's disease prevention and control system.

## Opportunities arising from the reform

### Improved emergency response capacity

The integration of disease surveillance and enforcement functions allows for more synchronized epidemiological investigation and regulatory actions. The newly structured disease prevention and control system facilitates real-time coordination between outbreak monitoring, emergency response and public health enforcement, ultimately reducing cross-departmental coordination costs and improving response efficiency.^2^

### Enhanced data sharing and smart surveillance

The consolidation of administrative functions has eliminated redundant expenditures and improved information exchange efficiency. A more streamlined data-sharing mechanism enables real-time monitoring, coordinated response and improved financial and logistical resource allocation. These advancements contribute to a more data-driven and proactive approach to public health governance.

## Challenges and future considerations

### Organizational and administrative adaptation

The CDC and Health Supervision Institutes historically operated with distinct administrative frameworks. The merger has led to transitional issues, such as unclear role definitions and decision-making conflicts. Establishing standardized operational procedures and clear role delineation will be crucial for ensuring the smooth functioning of the new system.

### Financial constraints

Despite the reform, public health expenditures remain low, accounting for <5% of China's total healthcare spending. Delays in funding allocation, budget shortages and difficulties in financial utilization have hindered operational efficiency. Additionally, inadequate investment in workforce remuneration and laboratory infrastructure remains a challenge. Sustainable financial support and effective budget monitoring mechanisms are essential for maintaining a resilient disease prevention and control system.

### Gaps in disease surveillance and digital infrastructure

Currently, infectious disease surveillance primarily relies on data from medical institutions, limiting its comprehensiveness. Information-sharing across agencies remains insufficient, creating barriers to timely epidemic response, and the government could allow for meaningful integration of evidence and knowledge from researchers, healthcare providers and practitioners at different agencies when designing policies.^3^ Leveraging big data, artificial intelligence and integrated digital platforms will be critical for enhancing China's disease surveillance and early warning systems.

China's disease control system reform represents a significant step toward strengthening public health governance. The integration of the CDC and Health Supervision Institutes has improved coordination, resource allocation and regulatory oversight. However, administrative restructuring, financial sustainability and digital infrastructure development remain key challenges. Addressing these issues through governance optimization, workforce capacity-building and technological innovation will be vital to ensuring an efficient and resilient disease prevention and control system.

## Data Availability

No new data were generated or analyzed in support of this study in the manuscript.
